# The PPAR-**γ** Agonist 15-Deoxy-**Δ** ^12,14^-Prostaglandin J_2_ Attenuates Microglial Production of IL-12 Family Cytokines: Potential Relevance to Alzheimer's Disease

**DOI:** 10.1155/2008/349185

**Published:** 2008-07-02

**Authors:** Jihong Xu, Steven W. Barger, Paul D. Drew

**Affiliations:** ^1^Department of Neurobiology and Developmental Sciences, University of Arkansas for Medical Sciences, 4301 W. Markham Street, Little Rock, AR 72205, USA; ^2^Department of Geriatrics, University of Arkansas for Medical Sciences, 4301 W. Markham Street, Little Rock, AR 72205, USA

## Abstract

Accumulation of amyloid-*β* peptide (A*β*) appears to contribute to the pathogenesis of Alzheimer's disease (AD). Therapeutic hope for the prevention or removal of A*β* deposits has been placed in strategies involving immunization against the A*β* peptide. Initial A*β* immunization studies in animal models of AD showed great promise. However, when this strategy was attempted in human subjects with AD, an unacceptable degree of meningoencephalitis occurred. It is generally believed that this adverse outcome resulted from a T-cell response to A*β*. Specifically, CD4^+^ Th1 and Th17 cells may contribute to severe CNS inflammation and limit the utility of A*β* immunization in the treatment of AD. Interleukin (IL)-12 and IL-23 play critical roles in the development of Th1 and Th17 cells, respectively. In the present study, A*β*_1−42_ synergistically elevated the expression of IL-12 and IL-23 triggered by inflammatory activation of microglia, and the peroxisome proliferator-activated receptor (PPAR)-*γ* agonist 15-deoxy-Δ^12,14^-PGJ_2_ (15d-PGJ_2_) effectively blocked the elevation of these proinflammatory cytokines. Furthermore, 15d-PGJ_2_ suppressed the A*β*-related synergistic induction of CD14, MyD88, and Toll-like receptor 2, molecules that play critical roles in neuroinflammatory conditions. Collectively, these studies suggest that PPAR-*γ* agonists may be effective in modulating the development of AD.

## 1. INTRODUCTION

Alzheimer’s disease (AD) is a neurodegenerative disorder and the most common cause of dementia in the elderly. AD is characterized by progressive memory deficits, changes in personality, and cognitive decline. It is believed that abnormal accumulation of amyloid-*β* peptide (A*β*), either as a soluble factor or as extracellular aggregates, contributes to the development of AD [1–3]. Cleavage of amyloid precursor protein (APP) can produce amyloid-*β* peptide 1-42 (A*β*_1−42_), the levels of which are correlated with neurotoxicity and development of AD. The connection between A*β* and AD symptoms is further strengthened by mouse models in which transgenic expression of the human A*β* precursor (APP) results in accumulation of A*β* and deficits in memory tests [4]. Preclinical investigations of anti-A*β* therapies have come to rely on such mice as a loose approximation of AD pathogenesis. The most successful anti-A*β* strategy demonstrated in these mice to date involves recruiting the immune system through vaccination. APP-transgenic mice that are immunized against A*β* at a young age never develop substantial A*β* deposits, and vaccination after deposition can even reverse a significant degree of the A*β* accumulation [5]. Most importantly, behavioral deficits are alleviated by such immunizations. These benefits correlate strongly with the titers of soluble antibody generated against A*β* [6–8], and passive immunization by injection of anti-A*β* antibody alone is also effective [9, 10]. Unfortunately, the first attempt to translate this vaccination approach to human AD patients generated iatrogenic meningoencephalitis in about 6% of individuals [11]. Mice can be induced to undergo similar reactions when overexpressing interferon (IFN)-*γ* [12], suggesting that immune responses tilted in favor of Th1 responses foster cell-mediated and/or inflammatory reactions to the vaccination. There is a considerable elaboration of inflammatory index in all AD brains [13, 14], including the activation of microglia; apparently, this neuroinflammation is fostered by A*β* itself [15, 16]. It is possible that these proinflammatory actions of A*β* create conditions unfavorable for the development of humoral immune responses.

IL-12 family cytokines are heterodimeric proteins which include IL-12 and IL-23. IL-12 is composed of p40 and p35 subunits, and IL-23 is composed of the same p40 subunit together with a unique p19 subunit [17]. IL-12 plays a critical role in the differentiation of CD4^+^ Th1 lymphocytes. These Th1 lymphocytes stimulate cell-mediated immune responses important in clearing pathogens, including viruses and bacteria. Th1 lymphocytes produce IFN-*γ* which activates cells of the innate immune system and contributes to the clearance of these pathogens. IL-23 stimulates the differentiation of a unique set of CD4^+^ T lymphocytes. These cells are characterized by the production of the cytokine IL-17 and are termed as Th17 lymphocytes [18]. Recent studies indicated that mice genetically ablated of the p19 subunit of IL-23 are resistant to the development of experimental autoimmune encephalomyelitis (EAE), whereas mice lacking the p35 subunit of IL-12 showed similar or more severe EAE than that observed in wild-type animals [19–21]. It thus appears that IL-12 and IL-23 each play important yet distinct roles in the development of immune responses that tend towards cell-mediated modalities which can include inflammation. Thus, suppressing the production of these cytokines may be effective in the treatment of inflammatory diseases.

Peroxisome proliferator-activated receptors (PPARs) are members of the nuclear hormone receptor family of transcriptional activators. Three PPAR subtypes exist (PPAR-*α*, PPAR-*γ*, and PPAR-*β*/*δ*), each exhibiting distinct patterns of tissue expression and ligand specificities [22]. The role of PPAR-*γ* in modulating adipogenesis and glucose metabolism is well established. Thiazolidinediones are PPAR-*γ* agonists that are currently used extensively in the treatment of type II diabetes. More recently, the role of PPAR-*γ* agonists in modulating immune responses, including immune responses in the CNS, has become appreciated. Nonsteroidal anti-inflammatory drugs (NSAIDs) have been shown to reduce AD risk and ameliorate microglial reactivity in AD brains [23]. Since NSAIDs bind to and activate PPAR-*γ*, resulting in reduced expression of proinflammatory genes, this receptor may mediate the observed anti-inflammatory effects of NSAIDs in AD brain. In addition, it has been demonstrated that the PPAR-*γ* agonists, pioglitazone and ibuprofen, reduced glial inflammation and A*β*_1−42_ levels in APPV717I transgenic mice [24]. Collectively, these studies suggest that PPAR-*γ* agonists may be effective in the treatment of neurodegenerative diseases, including AD.

Pattern recognition receptors termed as Toll-like receptors (TLRs) play a critical role in the innate immune response to pathogen-associated molecular patterns (PAMPs) present in viruses, bacteria, and fungi [25]. They may also contribute to neuroinflammation triggered by endogenous ligands [25] or simply overexpression of the receptors alone [26]. A series of eleven TLRs have been identified in mice and humans, each capable of binding distinct PAMPs. The PAMP lipopolysaccharide (LPS) binds to TLR4 in association with another pattern recognition receptor termed as CD14. With the exception of TLR3, ligand binding to TLRs stimulates recruitment of the adaptor molecule MyD88, activating a well-defined signal transduction pathway that culminates in activation of the transcription factor NF-*κ*B, which elevates expression of a variety of proinflammatory genes [27]. TLR4 has been suggested to play a role in regulating the pathogenesis of AD in humans [28, 29] and in animal models of AD [30]. This suggests that agents capable of altering MyD88-dependent TLR signaling may modulate the development of AD.

The current studies indicate that the PPAR-*γ* agonist 15d-PGJ_2_ suppresses the production of IL-12 and IL-23 by A*β* plus LPS-stimulated microglia. These cytokines play critical roles in Th1 and Th17 cell differentiation. These studies could have important implications concerning A*β* immunization as therapy for AD. In addition, we demonstrate that 15d-PGJ_2_ inhibits A*β* plus LPS stimulation of MyD88, CD14, and TLR2 expression by microglia, suggesting that this cyclopentenone prostaglandin inhibits MyD88-dependent signaling. This provides a potential mechanism by which the PPAR-*γ* agonist 15d-PGJ_2_ modulates the expression of proinflammatory cytokines.

## 2. MATERIALS AND METHODS

### 2.1. Reagents

15d-PGJ_2_ was obtained from Cayman Chemical Company (Ann Arbor, Mich, USA). Lipopolysaccharide and lectin, *Griffonia simplicifolia,* were obtained from Sigma (St. Louis, Mo, USA). A*β*_1−42_ was obtained from AnaSpec, Inc. (San Jose, Calif, USA). DMEM media, glutamine, trypsin, and antibiotics used for tissue culture were obtained from BioWhittaker (Walkersville, Md, USA). OPI medium supplement was obtained from Sigma. Fetal bovine serum (FBS) was obtained from Hyclone (Logan, Utah, USA). GM-CSF was obtained from BD Pharmingen (San Diego, Calif, USA). N-2 supplement was obtained from Gibco Invitrogen Corporation (Carlsbad, Calif, USA). Glial fibrillary acidic protein (GFAP) was obtained from Dako (Carpinteria, Calif, USA). C57BL/6 mice were obtained from Harlan (Indianapolis, Ind, USA) and bred in house.

### 2.2. Cell culture

Primary mouse microglia cultures were obtained through a modification of the McCarthy and deVellis protocol [31]. Briefly, cerebral cortices from 1–3 day-old C57BL/6 mice were excised, meninges removed, and cortices minced into small pieces. Cells were separated by trypsinization followed by trituration of cortical tissue. The cell suspension was filtered through a 70 *μ*m cell strainer to remove debris. Cells were centrifuged at 153 × g for 5minutes at 4°C, resuspended in DMEM medium containing 10% FBS, 1.4 mm L-glutamine, 100 U/mL penicillin, 0.1mg/mL streptomycin, OPI medium supplement, and 0.5 ng/mL recombinant mouse GM-CSF, and plated into tissue culture flasks. Cells were allowed to grow to confluency (7–10 days) at 37°C/5% CO_2_. Flasks were then shaken overnight (200 rpm at 37°C) in a temperature-controlled shaker to loosen microglia and oligodendrocytes from the more adherent astrocytes. These less adherent cells were plated for 2-3 hours and then lightly shaken to separate oligodendrocytes from the more adherent microglia. Microglia were seeded in 24-well plates or 6-well plates and incubated overnight at 37°C/5% CO_2_. After overnight incubation, cells were treated with 15d-PGJ_2_ for 1 hour in the serum free medium with N-2 supplement, and then stimulated with A*β*_1−42_ and/or LPS for 6 or 24 hours. A*β*_1−42_ peptides were dissolved in DMSO to prepare a 5 mM stock solution, which was aliquoted and stored at −80°C. A*β*_1−42_ stock solution was diluted with culture medium to a concentration of 0.1 mM, and set at room temperature for 12–18 hours before use. The final applied concentration of DMSO from A*β*_1−42_ was ≤0.2%. After the 24-hour stimulation, tissue culture supernatants were collected for enzyme-linked immunosorbent assay (ELISA), and cell viability was analyzed; 6 hours after stimulation, total RNA was collected for real-time quantitative RT-PCR (qRT-PCR) analysis. The purity of microglia cultures was greater than 95% as determined by immunohistochemical staining with the lectin, *Griffonia simplicifolia*. Astrocyte contamination of the microglial cultures was assessed by immunohistochemical staining with anti-GFAP.

### 2.3. Cell viability assay

Cell viability was determined by MTT reduction assay as described previously [32]. Optical densities were determined using a Spectromax 190 microplate reader (Molecular Devices, Sunnyvale, Calif, USA) at 570 nm. Results were reported as percent viability relative to untreated cultures.

### 2.4. Enzyme-linked immunosorbent assay (ELISA)

Cytokine (IL-12p40, IL-12p70, and IL-1*β*) levels in tissue culture media were determined by ELISA as described by the manufacturer (OptEIA Sets, Pharmingen, San Diego, Calif, USA). Cytokine IL-23 (p19/p40) levels in tissue culture media were determined by ELISA as described by the manufacturer (eBioscience, San Diego, Calif, USA). Optical densities were determined using a Spectromax 190 microplate reader (Molecular Devices, Sunnyvale, Calif, USA) at 450 nm. Cytokine concentrations in media were determined from standards containing known concentrations of the proteins.

### 2.5. RNA isolation and cDNA synthesis

Total RNA was isolated from microglia using the RNeasy Mini Kit (Qiagen Sciences, Md, USA). RNA samples were treated with DNAse1 (Invitrogen, Carlsbad, Calif, USA) to remove any traces of contaminating DNA. The reverse transcription (RT) reactions were carried out using an iScript cDNA synthesis kit (Bio-Rad, Hercules, Calif, USA) according to the manufacturer's instructions. 

### 2.6. Real-time quantitative RT-PCR assay

IL-12p40, IL-12p35, IL-23p19, IL-1*β*, CD14, MyD88, TLR2, and TLR4 mRNAs were quantified by real-time PCR using an iCycler IQ multicolor real-time PCR detection system (Bio-Rad). All primers and TaqMan MGB probes (FAMdye-labeled) were designed and synthesized by Applied Biosystems (Foster City, Calif, USA). The real-time PCR reactions were performed in a total volume of 25 *μ*L using an iCycler kit (Bio-Rad). The levels of IL-12p40, IL-12p35, IL-23p19, IL-1*β*, CD14, MyD88, TLR2, and TLR4 mRNA expression in primary microglia were calculated after normalizing cycle thresholds against the “housekeeping” gene GAPDH, and are presented as the fold induction value (2^−ΔΔCt^) relative to LPS-stimulated microglia.

### 2.7. Statistics

Data were analyzed by one-way ANOVA followed by a Bonferroni posthoc test to determine the significance of difference.

## 3. RESULTS

### 3.1. Effects of 15d-PGJ_**2**_ on IL-1*β* production by *β*-amyloid plus LPS-stimulated microglia

**Figure 1:**
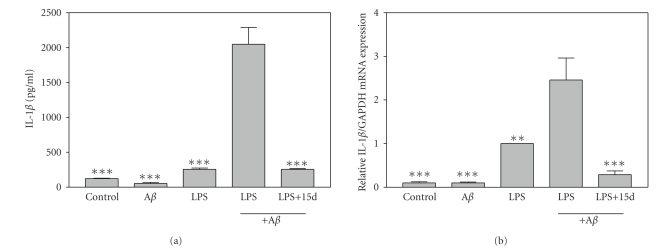
*15d-PGJ_2_ inhibits IL-1*β* expression by A*β*_1−42_ plus LPS-activated microglia*. (a) Primary mouse microglial cells were pretreated for 1 hour with 15d-PGJ_2_ (2.5 *μ*M). A*β*_1−42_ (5 *μ*M), LPS (10 ng/mL), or A*β*_1−42_ (5 *μ*M) plus LPS (10 ng/mL) was added as indicated, and 24 hours later, the concentration of IL-1*β* in the culture medium was determined. Values represent the mean ± s.e.m for a representative experiment run in triplicate. At least three independent experiments were conducted. (b) Cells were pretreated for 1 hour with 15d-PGJ_2_ (2.5 *μ*M). A*β*_1−42_ (10 *μ*M), LPS (5 ng/mL), or A*β*_1−42_ (10 *μ*M) plus LPS (5 ng/mL) was added as indicated, and 6 hours later, total RNA was isolated. IL-1*β* mRNA levels were determined by real-time quantitative RT-PCR. Results are expressed as fold inductions in GAPDH normalized mRNA values versus levels in LPS-treated cells. Values are mean ± s.e.m of six samples derived from three independent experiments, with each experiment performed in duplicate. **P < .01 and ***P < .001 versus A*β*_1−42_ + LPS-treated cultures.

A variety of studies suggest that the inflammatory cytokine IL-1*β* plays a significant role in modulating the pathogenesis of AD [33]. In the present study, we investigated whether A*β*_1−42_ plus a low dose of LPS could induce IL-1*β* production by primary mouse microglial cells. Our results showed that A*β*_1−42_ alone did not induce microglia production of IL-1*β* protein (Figure 1(a)) and IL-1*β* mRNA (Figure 1(b)). LPS (10 ng/mL) alone stimulated microglial production of IL-1*β* protein and mRNA, while a combination of A*β*_1−42_ and LPS synergistically induced the expression of IL-1*β* protein and mRNA. Interestingly, the PPAR-*γ* agonist 15d-PGJ_2_ strongly suppressed induction of IL-1*β* in A*β*_1−42_ plus LPS-stimulated primary microglial cells. The PPAR-*γ* agonist did not decrease the viability of these microglial cells compared to cells treated with A*β*_1−42_ plus LPS as determined by MTT analysis (data not shown). Therefore, the effects of 15d-PGJ_2_ on the production of IL-1*β* were not due to effects on cell viability. These studies suggest that 15d-PGJ_2_ may suppress the production of IL-1*β*, an inflammation-related cytokine associated with the pathogenesis of AD.

### 3.2. Effects of 15d-PGJ_**2**_ on IL-12 family cytokines by *β*-amyloid plus LPS-stimulated microglia

**Figure 2:**
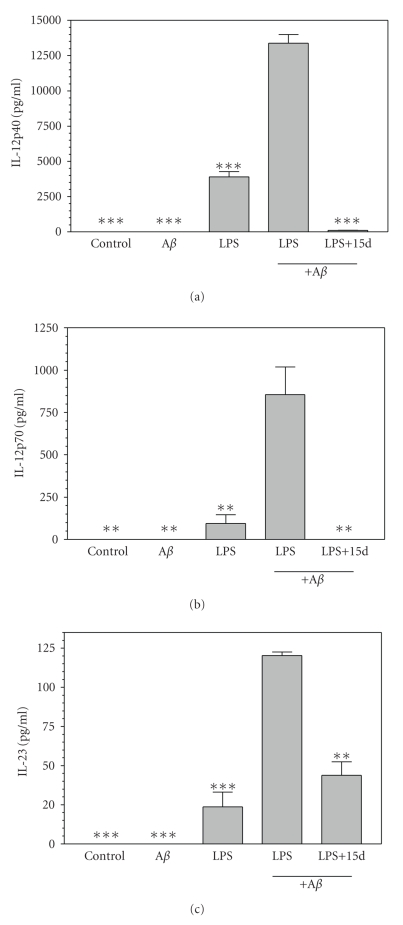
*15d-PGJ_2_ inhibits IL-12 family cytokines production by A*β*_1−42_ plus LPS-activated microglia*. Cells were pretreated for 1 hour with 15d-PGJ_2_ (2.5 *μ*M). A*β*_1−42_ (5 *μ*M), LPS (10 ng/mL), or A*β*_1−42_ (5 *μ*M) plus LPS (10 ng/mL) was added as indicated, and 24 hours later, the concentration of IL-12p40 (a), IL-12p70 (p35/p40) (b), and IL-23 (p19/p40) (c) in the culture medium was determined. Values represent the mean ± s.e.m for a representative experiment run in triplicate. At least three independent experiments were conducted. **P < .01 and ***P < .001 versus A*β*_1−42_ + LPS-treated cultures.

IL-12 family cytokines are believed to contribute to the differentiation of Th1 and Th17 cells. A*β*_1−42_ alone had little or no effect on the production of IL-12 family cytokines by microglia. LPS (10 ng/mL) stimulated microglia to secrete IL-12 family cytokines including IL-12p40 (Figure 2(a)), IL-12p70 (Figure 2(b)), and IL-23 (Figure 2(c)). In the context of this inflammatory priming, A*β*_1−42_ further increased microglial production of each of these IL-12 family proteins significantly. Furthermore, the PPAR-*γ* agonist 15d-PGJ_2_ significantly suppressed the expression of these IL-12 family proteins.

### 3.3. Effects of 15d-PGJ_**2**_ on expression of IL-12 family cytokine subunit mRNAs by *β*-amyloid plus LPS-stimulated microglia

**Figure 3:**
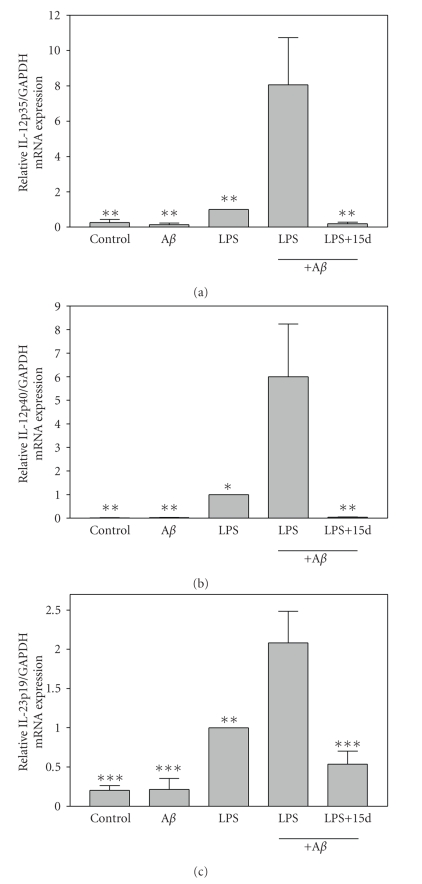
*15d-PGJ_2_ inhibits microglial mRNA expression of IL-12p40, IL-12p35, and IL-23p19 induced by A*β*_1−42_ plus LPS*. Cells were pretreated for 1 hour with 15d-PGJ_2_ (2.5 *μ*M). A*β*_1−42_ (10 *μ*M), LPS (5 ng/mL), or A*β*_1−42_ (10 *μ*M) plus LPS (5 ng/mL) was added as indicated, and 6 hours later, total RNA was isolated. IL-12p35 (a), IL-12p40 (b), and IL-23p19 (c) mRNA levels were determined by real-time quantitative RT-PCR. Results are expressed as fold inductions in GAPDH normalized mRNA values versus levels in LPS-treated cells. Values are mean ± s.e.m of six samples derived from three independent experiments, with each experiment performed in duplicate. *P < .05, **P < .01, and ***P < .001 versus A*β*_1−42_ + LPS-treated cultures.

A*β*_1−42_ alone had little or no effect on stimulating the expression of IL-12 family cytokine subunit mRNAs including IL-12p35 (Figure 3(a)), IL-12p40 (Figure 3(b)), and IL-23p19 (Figure 3(c)). Low doses of LPS (5 ng/mL) alone slightly induced the expression of these mRNAs. However, A*β*_1−42_ in combination with LPS elicited significantly higher levels of IL-12 family subunit mRNAs compared to microglia stimulated with LPS alone. Pretreatment with 15d-PGJ_2_ significantly suppressed the expression of IL-12 family subunit mRNAs. Thus, 15d-PGJ_2_ inhibits the expression of IL-12 family cytokines and the mRNAs that encode these proteins. IL-12 and IL-23 play critical roles in the differentiation of Th1 and Th17 cells, which may contribute to the inflammatory events that resulted in cessation of clinical trials involving immunization of A*β* in the treatment of AD. Thus, cotreatment with 15d-PGJ_2_ may increase the utility of A*β* immunotherapy for AD patients.

### 3.4. Effects of 15d-PGJ_**2**_ on expression of Toll-like receptor signaling

**Figure 4:**
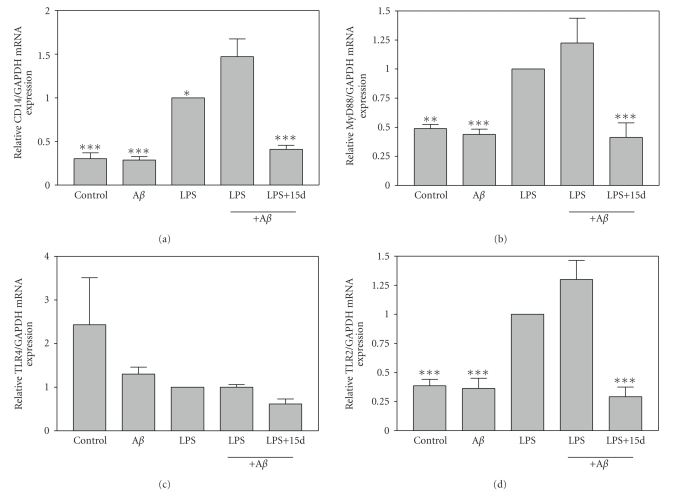
*The effects of 15d-PGJ_2_ on microglial mRNA expression of CD14, MyD88, TLR4, and TLR2 induced by A*β*_1−42_ plus LPS*. Cells were pretreated for 1 hour with 15d-PGJ_2_ (2.5 *μ*M). A*β*_1−42_ (10 *μ*M), LPS (5 ng/mL), or A*β*_1−42_ (10 *μ*M) plus LPS (5 ng/mL) was added as indicated, and 6 hours later, total RNA was isolated. CD14 (a), MyD88 (b), TLR4 (c), and TLR2 (d) mRNA levels were determined by real-time quantitative RT-PCR. Results are expressed as fold inductions in GAPDH normalized mRNA values versus levels in LPS-treated cells. Values are mean ± s.e.m of six samples derived from three independent experiments, with each experiment performed in duplicate. *P < .05, **P < .01, and ***P < .001 versus A*β*_1−42_ + LPS-treated cultures.

The MyD88-dependent TLR signaling pathway plays a critical role in modulating the response to PAMPs including LPS. We demonstrate that a combination of A*β*_1−42_ plus LPS significantly induced the expression of CD14 relative to microglia treated with LPS alone (Figure 4(a)). In addition, A*β*_1−42_ plus LPS also trended towards inducing MyD88 expression relative to each stimulus alone (Figure 4(b)). CD14 and MyD88 are critical intermediates in MyD88-dependent signaling. As we have demonstrated previously, LPS does not significantly induce the expression of TLR4, but does induce the expression of TLR2 [34]. Similarly, A*β*_1−42_ in combination with LPS did not induce microglial expression of TLR4 (Figure 4(c)), but did induce the expression of TLR2 (Figure 4(d)). Interestingly, 15d-PGJ_2_ inhibited A*β*_1−42_ plus LPS induction of MyD88, CD14, and TLR2 mRNA expression in microglia. These studies suggest that 15d-PGJ2 may suppress inflammatory responses stimulated by A*β*_1−42_ plus LPS by inhibiting MyD88-dependent TLR signaling.

## 4. DISCUSSION

AD currently affects over 200 million people worldwide. Disease incidence is expected to increase as the population ages, and the socioeconomic impact of AD is staggering. The disease is characterized in part by the presence of neuritic plaques which contain accumulations of insoluble A*β*. Vaccination with A*β* synthetic peptides in animal models of AD suggested that such immunizations may be effective in the treatment of AD in humans. For example, A*β* immunization of APP transgenic mice decreases the density and number of A*β* deposits in the brains of these mice. Decreased A*β* deposits in these mice are associated with decreased neuritic dystrophy and gliosis [7]. Intranasal administration of A*β* engenders humoral responses that include immunoglobulin isotypes consistent with a Th2 response, and this is associated with increased clearance of amyloid [35]. Significantly, active immunization against A*β* in APP transgenic mice decreases memory deficits in these mice [6, 35, 36]. Interestingly, passive administration of monoclonal antibodies specific for A*β* peptides is also effective in clearing A*β* and improving memory deficits in APP transgenic mice [37, 38]. This suggests that A*β*-specific antibodies produced following immunization are the critical factor mediating AD-like pathology in these animal models of AD. Three potential mechanisms have been suggested that may determine how anti-A*β* antibodies reduce A*β* deposits in the brains of APP transgenic mice. A*β* antibodies (1) may directly dissolve A*β* deposits, (2) may stimulate Fc-receptor-mediated phagocytosis of A*β* by microglia, and/or (3) may stimulate A*β* efflux from the brain to the plasma [3].

Animal studies indicating that A*β* immunization of APP transgenic mice reduced plaque burden in mice stimulated human clinical trials designed to evaluate the clinical efficacy of A*β* immunization in the treatment of AD. Small-scale phase I trials indicated apparent safety of A*β* immunization, and demonstrated that the majority of mild to moderate AD patients immunized in these studies produced anti-A*β* antibodies [39, 40]. However, subsequent larger-scale phase II clinical trials were halted when approximately 6% of A*β* immunized patients developed meningoencephalitis [11]. Postmortem evaluation indicated that A*β* immunization resulted in decreased plaque burden in the cortex of treated patients [41–43], and these brain regions were associated with abundant A*β* immunoreactive microglia, suggesting that these cells were involved in the removal of A*β* [43]. Interestingly, although anti-A*β* antibodies are believed to contribute to the reduction in A*β* plaques in AD patients, antibodies titers did not correlate with the development of meningoencephalitis [11, 40]. Several studies suggest that T cell responses to A*β* may have stimulated the development of meningoencephalitis in immunized AD patients [44–46]. Furthermore, a higher T cell reactivity to A*β* has been observed in some elderly and AD patients not immunized with A*β*. This suggests that the elderly population and AD patients may exhibit increased susceptibility to the development of meningoencephalitis following A*β* vaccination [46]. Cases of meningoencephalitis were associated with increased infiltration of both CD4^+^ and CD8^+^ T cells [42]. However, it is generally believed that CD4^+^ Th1 cells triggered the development of meningoencephalitis following A*β* immunization [3]. The potential role of the recently described CD4^+^Th17 cells in the production of meningoencephalitis has not been evaluated. However, studies indicating that these cells play a critical role in the development of MS and other autoimmune disorders suggest that these cells may also play a role in the development of meningoencephalitis in A*β* immunized AD patients.

Our current studies indicate that the PPAR-*γ* agonist 15d-PGJ_2_ inhibits microglial production of IL-12 and IL-23, which play critical roles in the differentiation of Th1 and Th17 cells, respectively. This suggests that 15d-PGJ_2_ could potentially increase the efficacy and safety of A*β* immunization of AD patients by decreasing or abolishing the development of meningoencephalitis in these patients. Epidemiological studies indicated that nonsteroidal anti-inflammatory drugs (NSAIDs) reduced the risk of AD. Some NSAIDs are capable of activating PPAR-*γ*, suggesting that these drugs may modulate development of AD through activation of this receptor [47, 48]. The role of PPAR-*γ* in modulating AD is supported by studies indicating that ibuprofen reduced A*β*_1−42_ levels in APP transgenic mouse models of AD, while low levels of the thiazolidinedione pioglitazone stimulated a slight yet statistically insignificant reduction of A*β*_1−42_ levels in these mice [49]. In a later study, higher levels of pioglitazone decreased astrocyte and microglial activation and A*β* plaque burden in APP transgenic mice [24]. Similarly, the thiazolidinedione rosiglitazone also decreased A*β*_1−42_ levels in animal models of AD [50]. Collectively, these studies support a role for PPAR-*γ* in modulating AD pathology. Studies indicate that PPAR-*γ* activation suppresses expression of *β*-site of APP cleaving enzyme (BACE)-1, suggesting that PPAR-*γ* agonists may modulate AD pathogenesis at least in part by altering A*β* homeostasis [51]. Importantly, recent clinical studies demonstrated that rosiglitazone was effective in improving cognition in a subset of AD patients [52, 53]. The fact that rosiglitazone exhibits poor blood-brain barrier penetration suggests that this PPAR-*γ* agonist may act in the periphery and not directly in the CNS.

We and others have previously demonstrated that PPAR-*γ* agonists are capable of suppressing the activation of NF-*κ*B, which is a potent transcriptional activator of a variety of genes encoding proinflammatory molecules. MyD88-dependent signaling results in the activation of NF-*κ*B. In the current studies, we demonstrate that the PPAR-*γ* agonist 15d-PGJ_2_ suppressed microglial expression of MyD88 and CD14 which are critical intermediates in MyD88-dependent TLR signaling. In addition, we demonstrate that 15d-PGJ_2_ inhibits microglial expression of IL-1*β*, a cytokine believed to contribute to AD pathogenesis [33]. Thus, PPAR-*γ* agonists may act as general suppressors of classical activation of microglia. Since classically activated microglia produce neurotoxic molecules, suppression of microglial activation may protect against AD. However, it should also be noted that some form of microglial activation may help remove A*β* plaques from AD brains through phagocytosis. In addition, TLR and CD14 molecules have been suggested to contribute to—or alternatively protect against—the development of AD [30, 54, 55]. It clearly appears that microglia and microglial products modulate AD through a series of complex and potentially conflicting mechanisms.

In summary, we have demonstrated that the PPAR-*γ* agonist 15d-PGJ_2_ inhibits production of IL-12 and IL-23 by A*β* plus LPS-activated microglia. These cytokines regulate the differentiation of Th1 and Th17 cells, which may limit the efficacy of A*β* immunotherapy for the treatment of AD. Furthermore, we demonstrate that 15d-PGJ_2_ inhibits the production of IL-1*β* by microglia, a cytokine known to play a role in AD pathogenesis. Finally, we demonstrate that 15d-PGJ_2_ inhibits the expression of MyD88-dependent signaling intermediates, suggesting a mechanism by which this PPAR-*γ* agonist may suppress inflammation. Collectively, these studies contribute to the body of evidence indicating that PPAR-*γ* agonists may be effective in the treatment of AD.
